# Supplementary material for: Integrated Transcriptomics Profiling in Chahua and Digao Chickens’ Breast for Assessment Molecular Mechanism of Meat Quality Traits
Source: Genes (Basel). 2022 Dec 28;14(1):95. doi: 10.3390/genes14010095 (PMC9859260; doi:10.3390/genes14010095)
Supplement: Supplementary file 1 [file genes-14-00095-s001.zip › genes-2008384-supplementary/Figures file.pdf]

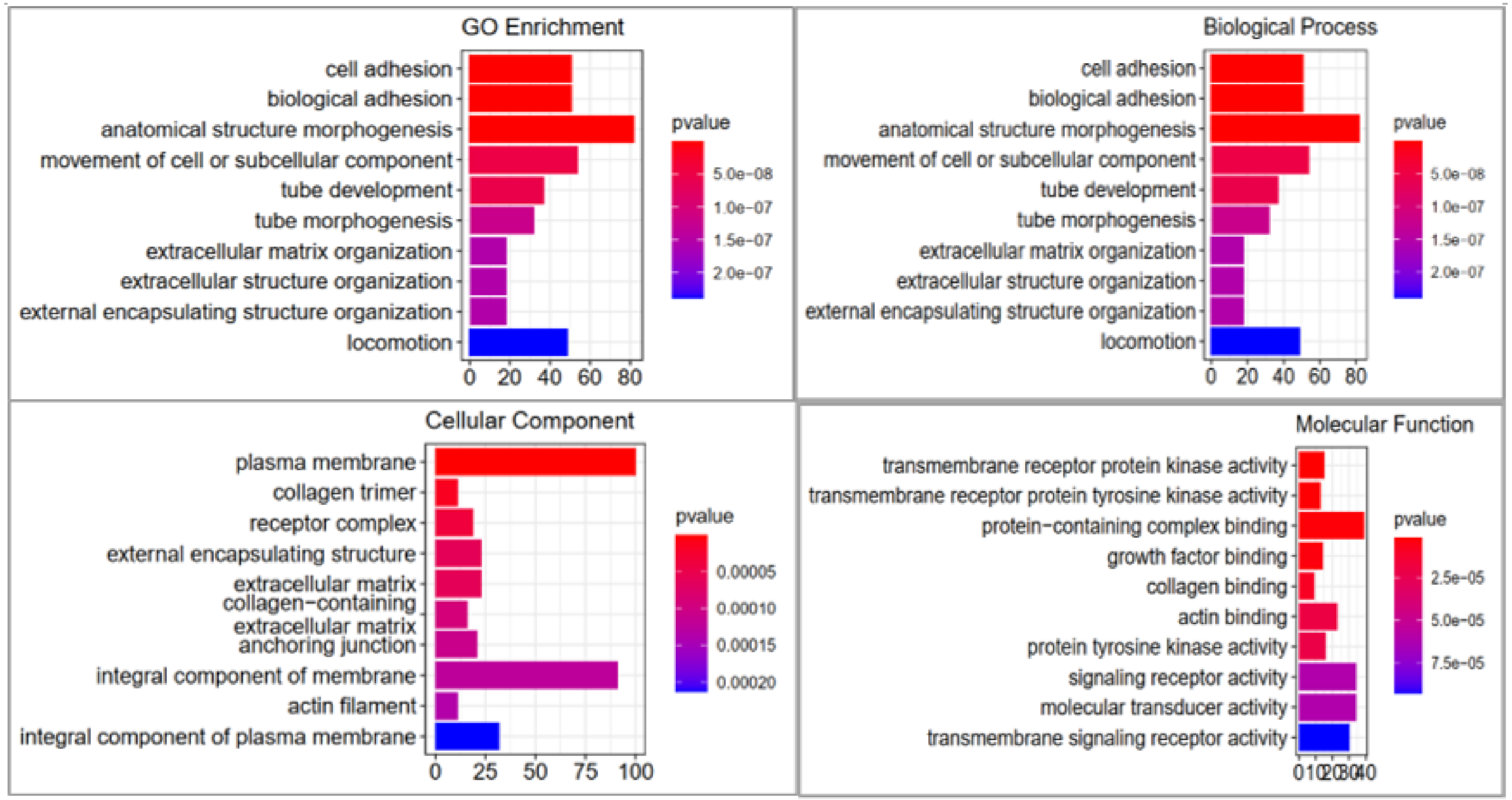

Figure S1. Histogram showing the GO functional terms of downregulated genes between CH and DG chicken.

In above left represents the top significantly enriched terms of all downregulated genes, in the above right represents the top significantly enriched terms of biological process downregulated genes. The down left represented the top significantly enriched terms of cellular component downregulated genes, and downright represented the top significantly enriched terms of molecular downregulated genes.

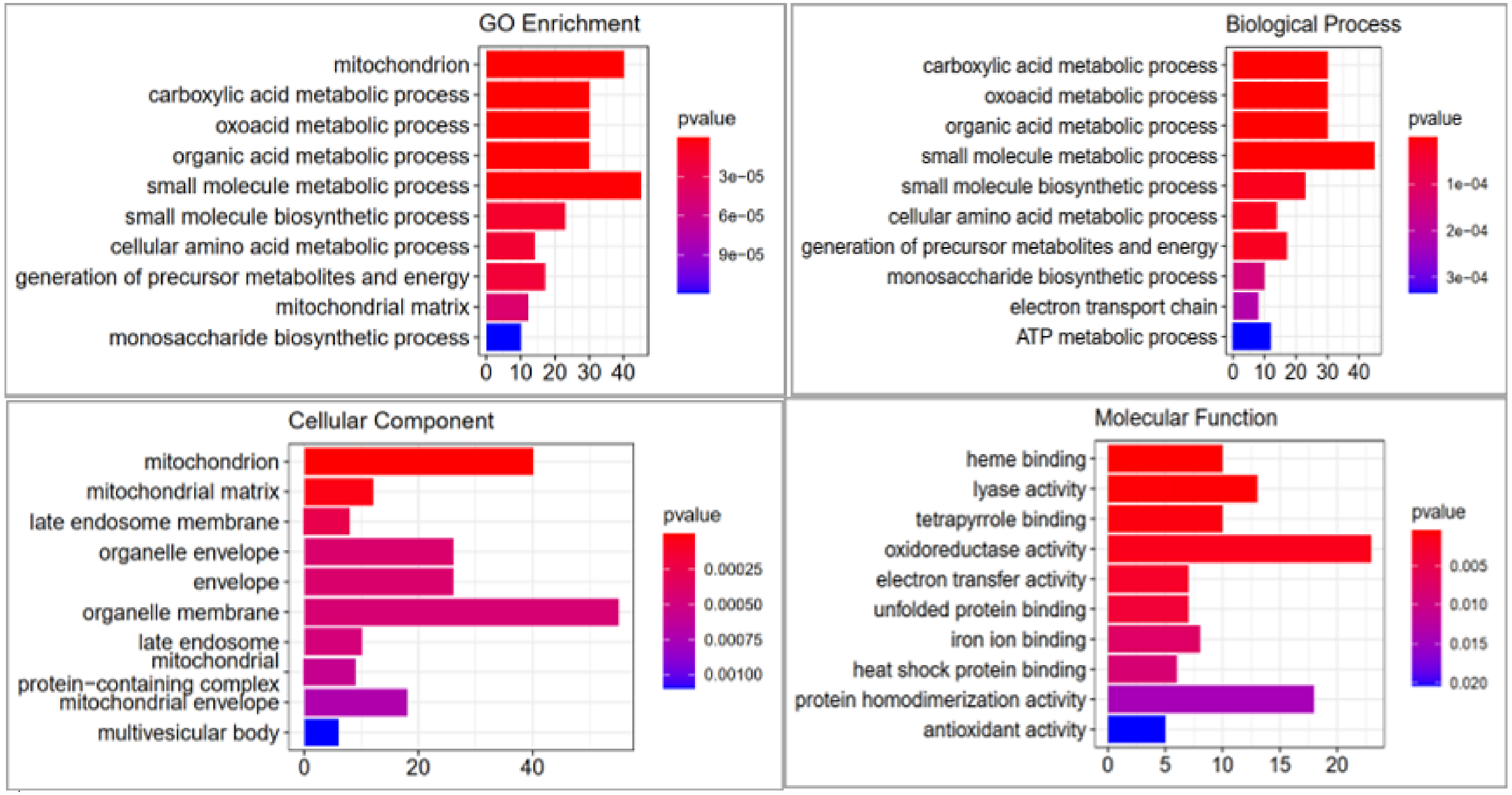

Figure S2. Histogram showing the GO functional terms of upregulated genes between CH and DG chicken.

The above left represents top significantly enriched terms of all upregulated genes, in the above right represents the top significantly enriched terms of biological process upregulated genes. The down left represented the top significantly enriched terms of cellular component upregulated genes, and in downright represented the top significantly enriched terms of molecular upregulated genes.

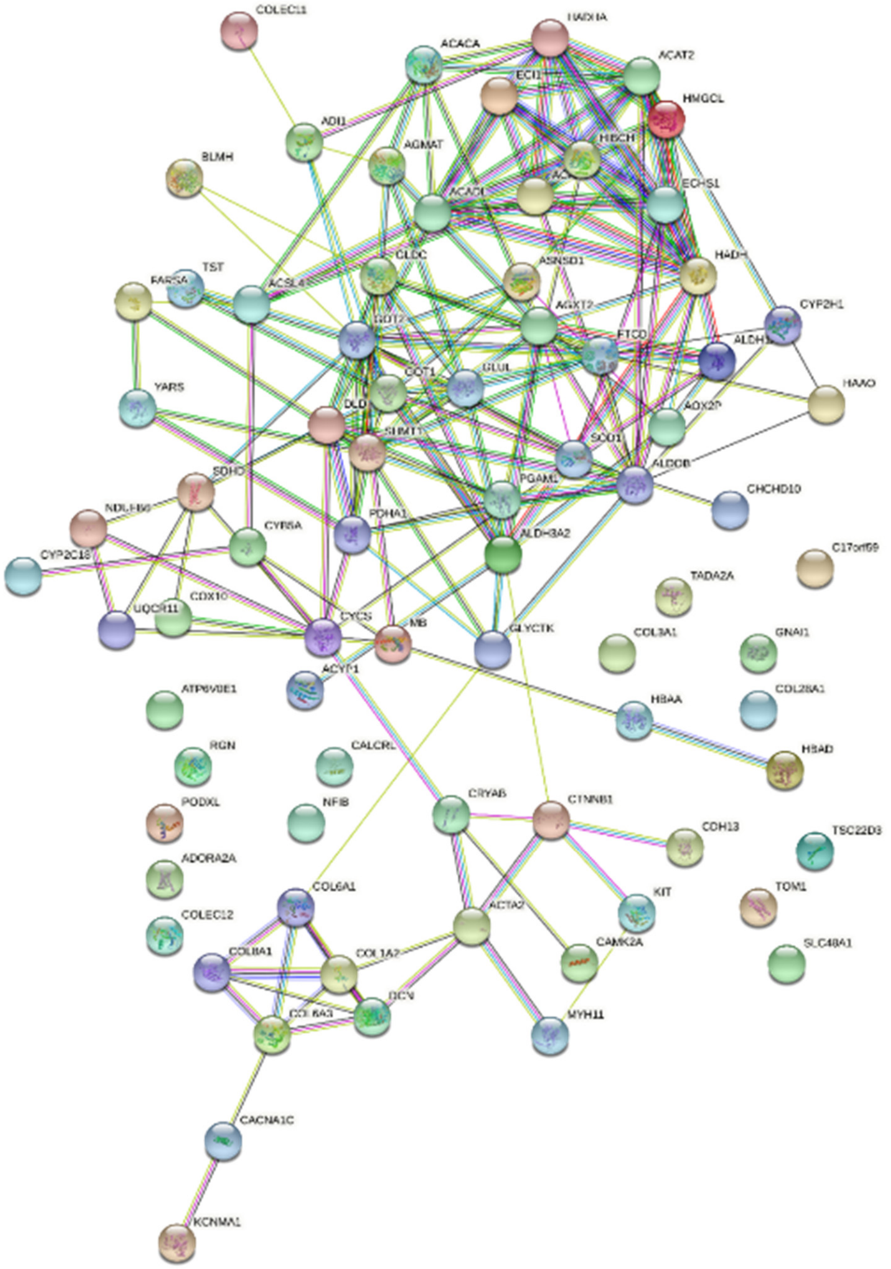

Figure S3. STRING network analysis on DEGs selected that may affect chicken meat traits
