# Supplementary material for: Integrated Transcriptomics Profiling in Chahua and Digao Chickens’ Breast for Assessment Molecular Mechanism of Meat Quality Traits
Source: Genes (Basel). 2022 Dec 28;14(1):95. doi: 10.3390/genes14010095 (PMC9859260; doi:10.3390/genes14010095)
Supplement: Supplementary file 1 [file genes-14-00095-s001.zip › genes-2008384-supplementary/Tables file.pdf]

Table S1. Composition of the Period I and Period II (g/kg, air dry) diets used in the experiment

Dietary Component	Period I[1]	Period II[1]
**Maize**	545.0	580.0
**Soy protein**	190.0	167.0
**Toasted soybean**	140.0	80.0
**Fish meal**	35.0	20.0
**Wheat bran**	30.0	100.0
**Soya oil**	25.0	18.0
**$CaHPO_4 \cdot 2H_2O$**	12.4	12.4
**Stone meal**	11.7	11.5
**Lysine**	2.2	2.2
**Methionine**	1.6	1.6
**Salt**	2.1	2.3
**Minerals and vitamins mix[2]**	5.0	5.0
**Metabolism Energy（MJ/Kg）**	12.8	12.6
**Crude protein (CP)**	205.5	183.5
**Crude fat**	54.1	56.5
**Calcium**	10.5	9.8
**Available phosphorus**	6.8	6.5
**Lysine**	13.5	12.1
**Methionine + Cysteine**	8.8	7.8

1: Period I is age 1-30 days; Period II is older than 30 days of age; 2: Supplied per kilogram of diet: antioxidant, 100 mg; biotin, 0.3 mg; vitamin A, 12,000 IU; vitamin D3, 3000 IU; vitamin E, 18.75 mg; vitamin K3, 2.65 mg; vitamin C, 12.6 mg; cyanocobalamin, 0.025 mg; folic acid, 2.2 mg; niacin, 35 mg; pyridoxine, 6 mg; riboflavin, 9 mg; thiamine, 3.0 mg; choline chloride, 600 mg; Co, 0.3 mg; Cu, 12 mg; Fe, 50 mg; I, 1 mg; Mn, 125 mg; Mo, 0.5 mg; Se, 200 µg; Zn, 60 mg.

Table S2 Primer information of DEGs.

Genes	Gene accession number		Sequence (5'->3')	Length	Tm (˚C)	GC%	Product length (bp)
**β-actin**	L08165.1	F	GTGTGATGGTTGGTATGGGC	20	58	59	225
		R	CTCTGTTGGCTTTGGGGTTC	20	58	58	
**COL28A1**	XM_040666104.1	F	AGTCCCTGGCATTGGAGAGC	20	61.9	60	112
		R	GCCACGTGGTCCTGTCATTC	20	61.3	60	
**COL1A2**	NM_001079714.2	F	GTAGGGTTGGGCCAATCGGT	20	62.2	60	193
		R	TTGCCAGTGACACCAGGAGG	20	62.0	60	
**MB**	NM_001167752.3	F	GCAACCGCCATAGTCAGCAC	20	61.98	60	136
		R	GTCCATGGCCAGCAATGTCG	20	62.00	60	
**CHCHD10**	XM_040684787.1	F	GGCCGTGCCACTACGAGAT	19	62.0	63.1	81
		R	CGTTGAAGCCCTCGCACAAG	20	62.1	60	
**FASN**	NM_205155.4	F	GCATGCTGGGCATGGAGTTC	20	62.0	60	170
		R	GGCATAAACCACAGGCACCG	20	61.9	60	
**IGF2**	XM_015286525.3	F	CCTTCGTGCCACTGCCTACC	20	63.1	65	171
		R	AGCACTCACATCCCAGGCAC	20	62.4	60	
**GHR**	NM_001001293.2	R	CCGAGTCCGATCAAGACAACG	21	61.0	57	157
		F	CAGCCCACACACTCCGAAGA	20	62.1	60	
**MYOD1**	NM_204214.3	F	GCAACGCCATCCGCTACATC	20	62.1	60	141
		R	ACTCCATCATGCCGTCGGAG	20	62	60	
**MYH10**	NM_205474.2	F	CAGCGATCTGGGCAGGAAGA	20	61	60	106
		R	TCCACACCAGCTTCTTGGCA	20	61	55	
**SMYD1**	NM_001397430.1	F	TGCAAGCTGTTGGTGTGGGA	20	62.5	55	103
		R	CGCAGCTCAATCTTGCCGTT	20	61.9	55	

***Table S3.* Significant enrichment of upregulated GO pathways associated with meat quality**

ID	Type	Description	p- value	p. adjusts	q- value	Gene ID	Count
**Down regulated pathways**							
GO:0005581	CC	collagen trimer	1.54E-05	0.00179	0.0014	COL6A3/COL6A1/COL1A2/LUM/COLEC12/COL8A1/COLEC10/COL3A1/COL14A1/COL6A2/COLEC11	11
**Upregulated pathways**							
GO:0006520	BP	cellular amino acid metabolic process	2.04E-05	0.00488	0.004813	GOT2/YARS/HMGCL/BLMH/FTCD/GOT1/TST/GLDC/ADI1/AGMAT/FARSA/ASNSD1/HIBCH/GLUL	14
GO:0009063	BP	cellular amino acid catabolic process	0.00039	0.05094	0.05022	GOT2/HMGCL/BLMH/FTCD/GLDC/HIBCH	6
GO:0020037	MF	*heme binding*	*0.00053*	*0.0053*	*0.0053*	*CYP2C23b/SDHD/CYCS/MB/CYB5A/HBAD/SUOX/SLC48A1/HBA1/CYP2C23a*	10

Table S4. Significant enrichment of down regulated KEGG pathways associated with meat quality

ID	*Description*	*Gene Ratio*	*Bg Ratio*	*p- value*	*p. adjusts*	*q- value*	*Gene ID*	*Count*
gga04512	*ECM-receptor interaction*	*27/547*	*84/5041*	*9.33E-08*	*4.86E-07*	*5.65E-06*	*396420/396133/396548/424442/428148/427355/396000/374058/396478/396076/415560/427850/396243/421749/428524/421960/418752/419031/420757/418893/396226/429806/101749098/395530/396538/396292/424121*	*27*
gga04020	*Calcium signaling pathway*	*45/547*	*225/5041*	*2.57E-05*	*0.00059*	*0.00051*	*395707/396107/419934/419175/428149/374175/395742/420416/427705/422330/395412/395760/424548/416730/395469/396259/373908/396364/421508/396413/408035/374128/396157/395891/420935/395323/395509/418212/427552/427212/427262/422013/374100/418978/396337/427635/770488/396446/374174/415805/396494/425224/420752/428279/428071*	*45*
gga04270	*Vascular smooth muscle contraction*	*26/547*	*122/5041*	*0.00048*	*0.0075*	*0.0065*	*419559/396465/423990/374065/423037/423787/396211/427705/395412/416730/421409/373908/396020/420854/395891/420935/418212/373970/427262/422013/771678/395340/373965/420786/768509/396469*	*26*
gga04330	*Notch signaling pathway*	*14/547*	*55/5041*	*0.0017*	*0.022*	*0.019*	*395128/395655/374031/416815/423208/396043/423958/395820/776138/418042/422802/427566/771101/395790*	*14*
gga00562	*Inositol phosphate metabolism*	*16/547*	*70/5041*	*0.0027*	*0.031*	*0.027*	*419999/419444/395356/419175/420416/423070/422312/424114/416730/422454/418690/415805/418498/424745/425224/428279*	*16*
gga04916	*Melanogenesis*	*19/547*	*91/5041*	*0.0034*	*0.0345*	*0.0300*	*374175/396368/395099/395508/416730/374062/396028/420854/418123/420935/427262/422013/373887/395340/374174/395703/374060/395964/378783*	*19*

Table S5. **Significant enrichment of upregulated KEGG pathways associated with meat quality**

ID	Description	Gene Ratio	Bg Ratio	p- value	p. adjusts	q- value	Gene ID	Count
gga00190	Oxidative phosphorylation	52/635	117/5041	5.21E-18	7.66E-16	6.74E-16	770135/417329/416391/424349/771510/416543/423763/415826/416013/416571/416978/419793/770937/423179/772150/424978/423280/770724/424575/422539/420624/418162/395758/769735/421939/419039/771939/418541/415752/772135/770638/431629/416336/420236/418118/420259/427897/420243/421392/107050898/426673/100859320/769492/768860/418104/417112/100859720/100857596/420337/430210/768854/100859641	52
gga01200	Carbon metabolism	36/635	99/5041	8.52E-10	6.26E-08	5.51E-08	417517/396456/396533/416520/396261/428969/419793/417699/395833/421281/421587/374009/427453/395217/395218/395758/396435/374222/427308/374189/422931/418610/395480/418856/418857/770828/423600/100858903/374064/374044/426629/100859720/423979/374263/427577/100859641	36
gga00280	Valine, leucine and isoleucine degradation	19/635	44/5041	4.06E-07	1.49E-05	1.31E-05	419739/424380/431666/396316/417615/395374/416642/417699/421587/420290/395929/424072/427269/770828/426847/420632/425758/423011/423979	19
gga00071	Fatty acid degradation	13/635	34/5041	0.000131	0.0027	0.0024	424005/417615/422345/422547/421587/420878/420290/395929/395979/770828/426847/416657/771098	13
gga00630	Glyoxylate and dicarboxylate metabolism	13/635	31/5041	4.18E-05	0.0012	0.0010	769005/417517/416520/426806/417699/421281/421587/374009/374222/423600/100858903/396489/427577	13
gga00020	Citrate cycle (TCA cycle)	12/635	28/5041	6.41E-05	0.0015	0.0013	417517/419793/417699/421281/374009/395758/418610/418857/100858903/100859720/374263/100859641	12
gga04146	Peroxisome	22/635	79/5041	0.00019	0.0036	0.0031	417526/100858408/396316/419303/422345/423201/420533/422547/374042/420878/421687/395938/421999/426247/423600/426601/769879/100857696/101751055/420131/416709/101747962	22
gga00620	Pyruvate metabolism	13/635	38/5041	0.00047	0.0077	0.0068	417517/396456/417615/416537/426806/417699/421281/423361/421587/374189/418610/395979/374263	13

gga00380	Tryptophan metabolism	12/635	37/5041	0.0013	0.0195	0.017	769005/417615/424041/417699/421396/421587/420290/395929/424072/770828/423600/771098	12
gga00030	Pentose phosphate pathway	9/635	25/5041	0.0023	0.028	0.025	421418/427453/395217/395218/427308/422931/395480/374064/427577	9
gga00010	Glycolysis / Gluconeogenesis	14/635	52/5041	0.0039	0.0445	0.0392	396456/417615/428969/417699/395833/395217/395218/396435/427308/418610/395979/374064/374044/100859356	14
